# Advances in vaccine development and the immune response against toxoplasmosis in sheep and goats

**DOI:** 10.3389/fvets.2022.951584

**Published:** 2022-08-25

**Authors:** Tanjila Hasan, Yoshifumi Nishikawa

**Affiliations:** ^1^National Research Center for Protozoan Diseases, Obihiro University of Agriculture and Veterinary Medicine, Obihiro, Japan; ^2^Department of Medicine and Surgery, Faculty of Veterinary Medicine, Chattogram Veterinary and Animal Sciences University, Chattogram, Bangladesh

**Keywords:** *T. gondii*, toxoplasmosis, vaccine, immune response, small ruminants

## Abstract

Toxoplasmosis is a zoonotic, parasitic infection caused by the intracellular, apicomplexan parasite *Toxoplasma gondii*, which infects all homeothermic animals including humans. The parasite has a major economic impact on the livestock industry. This is especially true for small ruminants (sheep, goats) as it is one of the most likely reasons for reproductive disorders in these animals. Primary infection in sheep and goats can result in a fetus that is mummified or macerated, fetal embryonic death, abortion, stillbirth, or the postnatal death of neonates, all of which threaten sheep and goat rearing globally. Humans can also become infected by ingesting bradyzoite-containing chevon or mutton, or the contaminated milk of sheep or goats, highlighting the zoonotic significance of this parasite. This article reviews the advances in vaccine development over recent decades and our current understanding of the immune response to toxoplasmosis in small ruminants (sheep, and goats).

## Introduction

*Toxoplasma gondii* is an obligate, single-celled, apicomplexan, protozoan parasite, responsible for toxoplasmosis in humans, livestock, and marine animals (1). Globally, it is considered one of the most successful parasites, infecting all warm-blooded mammals. It has been estimated that one-third of the world's human population is infected by this parasite, with prevalence ranging from ~10 to 50% depending on the geographical area (2). The disease is asymptomatic or exhibits non-specific flu-like symptoms in immunocompetent individuals (3), whereas it appears severe, or even fatal, in immunocompromised individuals (4). In pregnant women, the parasite can be transmitted *via* the placenta, in some cases leading to abortion of the fetus or even death of the mother and fetus (5, 6). According to the Center for Disease Control and Prevention (CDC), it is one of the identified foodborne zoonotic parasites that may cause human death (7).

This parasite presents a major challenge to the livestock industry as it is responsible for the reproductive failure of farm animals, such as stillbirths, abortions, postnatal mortality, and fetal malformations (8). It is prevalent among livestock, especially small ruminants such as sheep and goats (9). Sheep and goats are reared for milk, meat, and fiber, which significantly contribute to the economy of many countries (10, 11). It is reported that *T. gondii* causes congenital disease worldwide in sheep and goats (12). The veterinary surveillance study in the United Kingdom (UK) reported that *T. gondii* caused 28% of ovine abortions from 2014 to 2018 (13). The occurrence of clinical toxoplasmosis in sheep in the UK is between 1 and 2% per year (14) and if the rate of incidence is similar in Europe, which has around 66 million breeding ewes, the estimated losses of lambs due to toxoplasmosis could be ~1,320,000 per year. One report estimated losses of 70 million dollars because of toxoplasmosis in Australia (15). Recently, Gutiérrez-Expósito et al. (16) measured the direct financial losses happen due to an outbreak caused by *T. gondii* abortion in Spanish sheep flocks, and the estimated total direct economic costs were €5,154.5 (€171.8/abortion) in the dairy flock and €4,456 (€63.6/abortion) in the meat flock. Therefore, it is clear that *T. gondii* has a significant impact on the welfare of livestock and on the economy of the farming industry worldwide.

Felids are the only host species in which the sexual life cycle of the parasite occurs, and they also act as a definitive host (1). They can become infected following ingestion of *T. gondii*-infected wild birds or rodents. After ingestion by felids, tissue cysts containing *T. gondii* bradyzoites are degraded by different digestive enzymes within the gut of felids resulting in rupture of the cyst wall and release of bradyzoites. The bradyzoites invade the intestinal epithelium and develop within the epithelial cells. Within 2 days, bradyzoites move through all five schizont stages in the felid's intestinal epithelial cells and then transform into merozoites, the first sexual stage. The merozoites then divide into macrogametes and microgametes, which are fertilized to produce a zygote (1). Oocysts form from zygotes and are excreted in the environment with the feces of felids. Each oocyst possesses an impermeable outer shell that aids its survival in an adverse environment (17). Oocysts are typically non-pathogenic to other hosts until sporulation occurs. Within a few days, oocysts are sporulated in a suitable environment to generate sporozoites (18). Each sporulated oocyst contains two sporocysts, each comprising four sporozoites. Intermediate hosts (and definitive hosts as well) can get the infection through the ingestion of sporulated oocysts. Cats can discharge millions of oocysts into the environment which after sporulation can infect the intermediate hosts (19). It has been reported that 1–10 oocysts are required to infect a mouse (20, 21), 10 oocysts- to infect a sheep (21), and 100 oocysts to infect a cat (22). After ingestion of sporulated oocysts by intermediate hosts, sporozoites are released from the oocysts at the duodenal lumen of the host and are transformed into tachyzoites for a multiplication phase, possibly in the mesenteric lymph nodes. *T. gondii* tachyzoites can enter most nucleated cells of hosts and form a parasitophorous vacuole in which they multiply *via* an endodyogenous mechanism until they egress. Within the host cells, a transition from tachyzoite to bradyzoite occurs. This metamorphosis enables the parasite to evade the host's immune system (23, 24).

In a small ruminant, acute toxoplasmosis is manifested by a brief episode of fever, apathy, anorexia, diarrhea, and coughing (1, 9). In pregnant sheep species, the tachyzoites invade and proliferate within the tissues of the feto-maternal junction (25), resulting in fetal abortion, mummification, maceration, stillbirth, premature birth, or the delivery of a weak lamb that fails to survive long after birth (26, 27). *T. gondii* typically causes early abortions in sheep that usually occur 1 month after infection (25) but recent studies have also described a clinical presentation of early abortions only 14 days after experimental infection (27, 28) while late, or classical abortion occurs 19–26 days post-infection (29). In their study, sheep were inoculated with 50 oocysts of M4 strain in early (40 days of gestation), mid (90 days of gestation), and late gestation (120 days of gestation), and abortion was observed in all groups. However, the distribution of parasite was controlled in a better way in early and mid-gestation with lesser lesions than in late gestation (29). On the other hand, the dissemination of parasites was faster in late gestation to the placenta and fetus causing abortion and the lesions found in the fetuses were more visible in late gestation than those caused by early gestation. The mechanisms that trigger these variations are still unknown, however, it has been hypothesized that modulation of the immune response that occurs during pregnancy might be responsible (30, 31). The histopathological findings involve leukoencephalomalacia in the brain of lambs (32), infarcts and thrombosis in the caruncular villi of the placentomes, and ischemic lesions of ewes during the acute phase of abortion as a consequence of hypoxic damage to the fetus resulting abortion (28). It has also been described that, in the ovine, early abortions (40 days of gestation) cause increased infiltration of macrophages in caruncular septa, whereas in late (120 days of gestation) abortions the placentas containing the parasite had an increase of T lymphocytes and macrophages primarily in the fetal cotyledons (33). It has also been reported that the differences in peripheral and placental immune responses following *T. gondii* infection at different gestational ages in sheep may play role in pathogenesis (34). The immune response is mediated by the interaction of Th1 (IFN-γ and TNF-α), Th2 (IL4), and Treg (IL10) cytokine. The severity of infection is determined by the stage of pregnancy (27). The fetus is immunologically weak during early pregnancy; hence, the consequences are most severe if infection occurs early. However, as the pregnancy progresses, the fetal immune system grows stronger, as a result of which the clinical effects are less severe (27).

In 2005, a *Toxoplasma*-induced abortion storm was suspected among sheep in the United States (8). In the case of goats, a severe outbreak of clinical toxoplasmosis was documented in Brazil and Parana (35). The farming industry suffers financial losses as a result of morbidity and mortality among lambs and kids (10). Practically, it is difficult to assess the real losses caused by *Toxoplasma* in the ovine industry as the disease appears to be unpredictable, few aborted fetal samples are available for diagnosis, the samples that are submitted are often not adequately examined, and some submitted samples are not appropriate for diagnostic evaluation, and non-specific serodiagnosis occurs (10). Thus, this parasite is not only a public health burden but also an economic burden on the ovine industry (11).

To alleviate this economic burden, it is necessary to control the acute and congenital transmission of *T. gondii*. Vaccination might be one of the effective strategies to prevent abortion, vertical transmission, as well as acute and chronic infection, and tissue cyst formation. Effective vaccination could thereby help to protect sheep and goats and to prevent the risk of zoonosis. Our review article describes the *T. gondii* vaccines currently available for sheep and goats and explores our understanding of the immune responses triggered by these vaccines.

## Vaccines against *T. gondii* in sheep and goats and the associated immune response

Different types of available vaccines against *T. gondii* in sheep and goats and their characteristics are summarized in Table 1 and detail of these vaccines and the immune responses induced in vaccinated animals are described below.

**Table 1:** Characteristics of the developed vaccines against toxoplasmosis in sheep and goats.

**Sheep/goat**	**Type of vaccine**	**Vaccine**	**Dose**	**Adjuvant**	**Challenge**	**Route of vaccination**	**No. of an animal immunized/non-immunized**	**Results**	**References**
Sheep	Killed	Tachyzoites of RH strain	1^st^ dose−50 μg 2^nd^ dose−30 μg	ISCOMs	Tachyzoites of C56 ME49 oocysts	S. C	5 immunized, 5 non-immunized (fed oocyst orally), and 2 controls	Protection was significantly observable against tachyzoites and oocysts stage	(36)
	Killed	Tachyzoites of RH strain (soluble antigens)	200 μg	PLGs, CT	Oocysts of an M3 strain	Intranasal	-	Production of profuse immune response locally and systemically and production of anti-mucosal IgA	(37)
	Attenuated	Tachyzoites of S48 strain (in a modified form)	Prior mating−10^5^ tachyzoites/ewe	-	-	I. M	64 immunized, 30 non-immunized, 10 vaccinated control, and 10 unvaccinated control	The survival percent of alive lambs delivered by the immunized dam was increased by more than 70% in comparison to non-immunized dam	(38)
			Prior mating−10^5^ tachyzoites/ewe			S. C		The survival rate of offspring from a vaccinated dam lengthened up to 68% than the non-vaccinated one	
	Attenuated	ME-49 strain	10^6^ oocysts	-	4 × 10^6^ oocysts M3 strain	-	9 immunized lamb, 4 non-immunized lamb	Could not protect against the establishment of cysts in the tissue. All the mice died who have fed muscle specimens of non-immunized lamb	(39)
	Attenuated	Mic1-3KO from tachyzoites of RH strain ME49 oocysts	Prior mating−10^5^ tachyzoites/ewe		PRU oocysts	S. C	71 immunized ewes, 34 non-immunized controls	The survival percent of alive offspring increased between 62–91%	(40)
			Prior mating−10^5^tachyzoites/ewe			S. C		The survival percent of alive offspring increased up to 71%	
			Prior mating−10^5^tachyzoites/ewe			I. P		Induced IgG serum antibody response	
								The survival percent of alive offspring increased up to 92%	
	Live	incomplete S48 strain	10^5^ tachyzoites of the S48 strain/sheep	-	M4 oocyst		10 immunized and 2 non-immunized controls in two groups of sheep	Could not protect against the establishment of tissue cysts	(41)
	DNA vaccine	GRA1, GRA4, GRA6, and GRA7	1 mg of each plasmid	Liposomes	-	-	48 immunized (4 groups) and 12 non-immunized controls (2 groups)	Production of cell mediate and humoral immunity	(42)
	DNA vaccine	GRA7	1 mg of plasmid	Liposomes, Emulsigen P, Emulsigen D	-	-	36 immunized (3 groups), 24 non-immunized controls (2 groups)	The production of immune response enhanced significantly after using Liposomes and Emulsigen D as adjuvant	(43)
	DNA vaccine	MAG1	1 mg of plasmid	Liposomes	-	-	21 immunized animals (2 groups, 12 and 9 sheep respectively), 24 non-immunized controls (2 groups)	Strong antibody response	(44)
	DNA vaccine	pROP1, pSAG1 pROP1+ pSAG1	300 μg	Liposomes	-	I. M	40 sheep, 30 immunized (6 groups), 10 controls (2 groups)	Induced ROP1 -specific IgG1 and IgG2 antibodies significantly and IFN-γ production. In the case of SAG1, only SAG1-specific IgG1 and IgG2 production but no IFN-γ production	(45)
	DNA vaccine	MIC3	1 mg of plasmid	Liposomes	-	I. M	24 immunized, 12 non-immunized controls	Induced humoral response, production of Th1 and Th2 response after the booster dose	(46)
	DNA vaccine	ROP1-CD154	1 mg of plasmid	Liposomes	-	I. M	24 immunized (2 groups), 24 non-immunized controls	Strong cell-mediated and humoral immunity observed	(47)
	Recombinant vaccine	BCG producing GRA1	10^9^		2000, M3 strain	S. C	20 immunized, 8 non-immunized controls	Cell-mediated immune response only Partial protection	(48)
	Nano-particle	*Toxoplasma gondii* proteins (Total extract)	200 μg	Maltodextrin nano-particles	5 × 10^6^ tachyzoites of type I RH strain	Intranasal	36 immunized, 12 non-immunized controls	T helper 1- mediated immune response, production of IFN-g and IL-12. Protected against latent toxoplasmosis and prevent vertical transmission	(49)
Goat	Live	S48 strain	10^6^	-	1000 oocyst	I. M	17 immunized, 16 non-immunized controls	Decreased abortion rate	(50)
	Live	*Hammondia hammondi*	10^6^	-	*T. gondii* (GT1 strain)	Orally	6 immunized, 2 non-immunized controls	4 out of 5 does deliver healthy kids	(51)
	Live	*Hammondia hammondi*	10^3^–10^5^	-	*T. gondii* (GT1 strain)	Orally	12 immunized	Produced low level of ab against *T. gondii*	(52)
		*H. heydorni*	10^3^–10^5^					Production of no antibody	

### Live vaccines

Toxovax™ (Animal Health Ltd, New Zealand) was the first successful live attenuated vaccine to control congenital toxoplasmosis in sheep and goats (38, 53). S48, the vaccine strain, was first isolated in New Zealand from an aborted lamb. Later, this strain was maintained in the laboratory by a continuous passage in mice and eventually, after ~3,000 passages, lost its virulence. It failed to develop tissue cysts or oocysts (41) and was therefore unable to maintain prolonged infection in the host animal. Katzer et al. (41), vaccinated lambs with a live S48 strain, which is unable to form tissue cysts or oocysts, the main infective parasite stages, then challenged the animals with the M4 strain of *T. gondii* (Table 1). Lambs were euthanized at 2, 4, and 6-week post-challenge, and the DNA from parasites was detected in the heart and muscle tissues. The amount of parasite DNA was significantly lower in vaccinated lambs than in unvaccinated lambs. Moreover, the DNA of strain S48 was detected in muscle tissue and the lymph nodes of immunized lambs 6 weeks post-infection, showing that parasite DNA or tachyzoites may persist longer than previously anticipated even after immunization. These findings suggested that immunizing lambs with the S48 strain can reduce the development of tissue cysts after challenge infection, but fail to prevent tissue cyst formation.

In ewes, inoculation with 10^5^ tachyzoites of strain S48 induced both cellular and humoral immunity (4, 54), and after oral challenge with *T. gondii* oocysts conferred significant protection against abortion (54). When a naive sheep is inoculated with Toxovax™, the parasite starts to multiply in the local lymph nodes, resulting in a slight febrile response, and by 6 weeks the antibody titer peaks. The CD4^+^ and CD8^+^ T cells and the cytokine IFN-γ production were involved in the protective response induced by the vaccination in sheep (53). When a vaccinated pregnant ewe ingests oocysts on pasture, the sporozoites pierce the intestinal wall and enter the mesenteric lymph nodes, where the immune system prevents the parasite from spreading through the lymphatic system into the host's blood. Hence, the parasite will not be able to cause disease, and dissemination to other tissues will also be limited. Furthermore, vaccination not only reduces the abortion rate but also reduces the production of *T. gondii* tissue cysts in *T. gondii*-infected meat from sheep.

Reduction of the parasite burden in meat improves food safety and reduces the potential risk of transmission from sheep to humans (55). This is the only licensed vaccine for veterinary use, and a single dose is sufficient to induce immunity. One important consideration regarding vaccination is that 18 months after the initial dose, protective immunity can be induced that prevents abortion (54, 56). Therefore, sheep need to be immunized before mating, and a booster dose is needed 2 years later. In the case of milk and meat, 6 weeks is considered as the withdrawal period. The key advantage of employing a live vaccine is that it is comparatively easy to stimulate appropriate cellular immunity as the antigens are presented to the host in a similar manner to a natural infection. This may explain why the most successful vaccines used in the veterinary field to date are live vaccines. Although the Toxovax™ vaccine can partially protect immunized sheep against abortion, it represents potent disadvantages of live vaccines in terms of safety, production, and stability. As the genetic basis for its attenuation is unexplored, a possible reversion to virulence cannot be ignored. Moreover, the short viability of tachyzoites in an extracellular environment may largely hinder their production and maintenance in the long term (57). Live vaccines are not considered safe for human use, but the currently available live vaccine against *T. gondii* is the most effective option to date for use in sheep and goats. Research is ongoing to identify an effective alternative.

### Tachyzoite-based vaccines

To avoid the risks associated with live vaccines, over the past few decades, researchers have been developing a killed vaccine, with a prolonged shelf-life, for use in sheep. The killed vaccine contains extracts of tachyzoites but does not exhibit the same level of efficacy as a live vaccine. Lundén (36) used tachyzoites of *T. gondii* strain RH to prepare immune-stimulating complexes (ISCOMs) (Table 1). Sheep were vaccinated with the *Toxoplasma* ISCOMs subcutaneously and later challenged with *T. gondii* oocysts orally. After the first dose, the immunized sheep generated low levels of IgM and IgG. However, after the second dose, the IgG level was significantly higher, but there was no such increase in IgM in the vaccinated animals. The immunized animals also displayed a high IgG response after infection with sporulated oocysts. Stanley et al. (37) developed a mucosal immunization strategy that involved micro- and nano-particles consisting of poly (d,l-lactide-co-glycolide) (PLG) (Table 1) that were used to encircle the crude extract of *T. gondii* tachyzoites. Sheep were vaccinated with particulate *Toxoplasma* antigen *via* the intranasal route and were then challenged with sporulated oocysts orally. After mucosal vaccination, all of the animals produced higher levels of antigen-specific IgA antibody, which is indicative of a humoral immune response, as well as significant levels of IFN-γ, which is indicative of a cellular immune response. After the challenge, mucosal and systemic IgG levels increased significantly in the immunized sheep compared with the unimmunized controls, but the produced antibody was not sufficient enough to protect the animals from the challenge infection. In addition, the immunized animals showed a fever reaction to *Toxoplasma* infection. In another study, Falcón and Freyre (39) investigated the use of ME-49 strain to vaccinate sheep (Table 1). Lambs were immunized with 10^6^ oocysts of the ME-49 strain and were challenged with 4 × 10^6^ oocysts of the M3 strain. Complete protection was conferred against the production of cysts in the muscle tissue of lambs which had been immunized with the ME-49 strain before challenge infection, and this protection did not occur in unimmunized lambs. Mévélec et al. (40) investigated the effectiveness of a mutant RH strain, lacking the *mic1* (Mic1 knockout, KO) and *mic3* (Mic-3 KO) genes, (Table 1) in protecting against *T. gondii* miscarriage in sheep. At first, ewes were inoculated with 10^5^ Mic1-3 KO tachyzoites subcutaneously. Mic1-3 KO-injected ewes developed mild clinical indications of fever after vaccination, as well as the generation of serum antibodies, which remained stable throughout the testing period. Two months following immunization, ewes were mated and orally challenged with the PRU strain of *T. gondii* during the mid-gestational stage. When vaccinated pregnant ewes were challenged, they developed a slight fever response, but unvaccinated ewes developed a more severe, long-lasting febrile response. Furthermore, when challenged, all unimmunized ewes aborted, but 62% (Bizet ewes), 91% (Romanov ewes), and 64% (Sologonot ewes) of the lambs from immunized ewes survived and exhibited no clinical signs of disease. Mutant strain Mic1-3 KO performed equally well as S48, a live sheep vaccine strain (Toxovax).

### Vector-based vaccines

The use of a wide range of bacteria, viruses, and protozoa as vaccine vectors has become increasingly popular (58). One advantage of using a live vector vaccine is that it enables the effective presentation of antigen to T cells, plus it is safe to transfect a foreign DNA into the vector (59, 60).

Bacille Calmette-Guérin (BCG), an attenuated strain of *Mycobacterium bovis*, has been widely used for decades as a live vaccine with intrinsic adjuvant properties, especially for the induction of a cellular immune response (61). After immunization with recombinant BCG, mice developed cellular and humoral immune responses to various heterologous antigens (48, 61). Supply et al. (48) developed a recombinant BCG vaccine that expresses *T. gondii* dense granule protein 1 (TgGRA1) antigen (recBCG-TgGRA1) (Table 1). The recBCG-TgGRA1 vaccine was subcutaneously injected into sheep with a dose of 10^9^ BCG, and a booster dose was administered intravenously with 10^8^ BCG, 12 weeks after the first dose. Then, sheep were challenged with the M3 strain (2000 oocysts) of *T. gondii*. In immunized sheep, GRA1-specific cell-mediated immunity was induced, but not GRA1-specific humoral immunity. Moreover, sheep vaccinated with recBCG-TgGRA1 showed rapid onset of fever for a short duration, in comparison to control animals, which might suggest that early inflammatory response in association with immunity is faster in immunized animals than that of control ones. However, the recBCG-TgGRA1 vaccine was unable to produce sufficient protection against challenge infection.

### DNA-based vaccines

DNA-based vaccines have also been gaining popularity. DNA vaccines are easy to construct, and DNA vaccine vectors are inert, non-multiplying, and non-disseminating, which reduce the risk of pathogenic transformation. Furthermore, DNA vaccines can trigger antigen-specific T and B cell immune responses by inducing the expression of target antigens (62–64). Many researchers have established the effectiveness of DNA vaccination in human and animal models (65, 66). In clinical trials, DNA vaccination does not cause any noticeable side effects (66). Furthermore, DNA vaccines are easy to preserve, more heat-resistant than traditional vaccines, and can be produced in large quantities at low cost. The production of Th1 and Th2 immune responses makes DNA vaccines an ideal choice to combat *Toxoplasma* infection (67). Although DNA immunization has been shown to be effective in producing an immune response in small animals such as hamsters and mice, it has been less successful in inducing immunity in small ruminants such as sheep and goats. A number of different studies have been carried out on the use of DNA vaccines in sheep (42–44). Hiszczyńska-Sawicka et al. (43) tested several adjuvant formulations, including liposomes, emulsigen P, and emulsigen D, along with a DNA vaccine vector expressing the *T. gondii* dense granule protein 7 (TgGRA7) antigen, pVAXIgGRA7 (Table 1). Sheep were vaccinated intramuscularly at weeks 0 and 4 and the results indicated that immunization of sheep with plasmids formulated by liposomes and emulsigen D produced significantly higher levels of specific IgG1 and IgG2 than those immunized with emulsigen P plasmid formulations (43). Moreover, all of the vaccinated sheep produce significantly higher levels of IFN-γ than the control group, which suggested that a DNA vaccine formulated with liposomes and emulsigen D as an adjuvant, induced a strong immune response to *T. gondii* infection. In another study, Hiszczyńska-Sawicka et al. (42) tested DNA encoding four different dense granule proteins of *T. gondii* (i.e., TgGRA1, TgGRA4, TgGRA6, and TgGRA7) in conjunction with liposomes as an adjuvant, to evaluate the immune response to these candidate DNA vaccines. Sheep were inoculated twice, intramuscularly, with pGRA1, pGRA4, pGRA6, and pGRA7 vectors formulated into liposomes. After both the first and second doses, all of the vaccinated sheep evoked a cellular and humoral immune response, as evidenced by antibody and IFN-γ production. Among the different plasmids encoding antigens, pGRA7 was capable of inducing a significantly higher level of anti-GRA7-specific IgG2 and an IFN-γ immune response, which is indicative of a Th1 protective immune response, in non-pregnant ewes. However, vaccination with pGRA1, pGRA4, and pGRA6 induced an IgG1-type immune response with limited production of IFN-γ.

In a further study, Hiszczyńska-Sawicka et al. (44) used *T. gondii* matrix antigen 1 (TgMAG1) (found in the parasitophorous vacuolar matrix in tachyzoite vacuoles and the cyst wall and matrix in bradyzoite vacuoles) (Table 1) as a liposome-formulated DNA vaccine, with or without co-expressed ovine IL-6, to evaluate the anti-TgMAG1 immune response. Sheep were intramuscularly injected at weeks 0 and 4 with pMAG1, either alone or in combination with pIL-6. Sheep vaccinated with pMAG1 provoked significant levels of immunoglobulins IgG1 and IgG2, whereas no significant effect was observed on IgG1 and IgG2 levels in sheep vaccinated with pMAG1 co-expressed with IL-6, which indicated that IL-6 lacked adjuvant properties in this study. However, they did not perform challenge infection with *T. gondii* to confirm the protective efficacy of the vaccines, which might be a limitation of the study.

The micronemal protein, MIC3, a key adhesive protein that attaches to host cells and parasite membranes, has also been explored as a vaccine candidate by Hiszczyńska-Sawicka et al. (46) (Table 1). This MIC3 protein is found in all *T. gondii* infective stages, including tachyzoites, bradyzoites, and sporozoites (68). Using this protein, *T. gondii* can easily adhere to host cells in the early stages of infection, and it can also stimulate a robust immunological response in humans and mice (68, 69). Sheep were injected with pMIC3 formulated into liposomes *via* the intramuscular route. T helper 1 and T helper 2 immune responses against *Toxoplasma* infection were induced in vaccinated sheep, as evaluated by cellular (IFN-γ) and humoral (IgG1, IgG2, and anti-MIC3 antibody) immune responses. CD154 receptor is presented on dendritic cells (DCs) (70) and DCs are activated by the CD154 receptor, which regulates B cell growth (68). In the case of mice and sheep, it was reported that plasmid encoding CD154 succeeded in enhancing humoral and cellular immune responses (71, 72). In another report, in bovine, pCD154 combined with a shortened version of bovine herpes virus-1 glycoprotein D (tgD-CD154) elicited a tgD-CD154-specific immune response (72). Subsequently, in another study, a DNA vaccine encoding *T. gondii* rhoptry protein 1 (ROP1) antigen fused with ovine CD154 to evaluate the immune response against *Toxoplasma* infection in sheep (47) (Table 1). Sheep were intramuscularly inoculated with pROP1-CD154 and pROP1. In sheep vaccinated with pROP1-CD154, significant Th1 and Th2 immune responses were detected, whereas, in sheep vaccinated with pROP1 alone, only a Th1-specific immune response was detected. Furthermore, in another study (45), vaccination of sheep with plasmid encoding ROP1 greatly increased antigen-specific IgG1 and IgG2 antibodies in blood serum, and IFN-γ was produced when the *Toxoplasma* antigen was cultured in peripheral blood cells. These findings suggested that ROP1 may be an effective vaccine candidate to protect sheep against *Toxoplasma* infection. They also evaluated the immune response induced by pROP1 and pSAG1, but the protective efficacy of this vaccine was not confirmed by *T. gondii* challenge infection. Despite the fact that DNA immunization elicits distinct humoral immune reactions and cytokine secretion, the protection provided by DNA vaccines remains to be confirmed.

### Nanoparticle-based vaccine

In recent years, the application of nanoparticles has been considered to transport *T. gondii* antigens through the mucosal channel to mimic natural transmission. Ducournau et al. (49) studied the protective efficacy of nanoparticles consisting of maltodextrin with a phospholipid base (DGNP) (Table 1) as a vaccine candidate against latent and congenital *T. gondii* infection in sheep. Vaccinated sheep elicited a distinct Th1 cellular immune response after two intranasal or intradermal doses, as indicated by the production of IFN-γ and IL-12. In intradermally-vaccinated sheep, IL-10 secretion appeared to modulate this Th1 response, whereas it was lacking in sheep immunized *via* the intranasal route. Intranasal immunization resulted in a considerable reduction in brain cysts in immunized sheep (0/11) compared with unimmunized sheep (5/8) after challenge infection. Later, the brain cysts of newly borne lambs were counted and only one lamb out of eight was found to be positive (12%) from the intranasally vaccinated group than control (5/8, 62.5%). In the case of lambs, although *Toxoplasma* DNA was detected in almost all brains, cysts were not detected by direct counting in the brains of lambs from nasal immunized ewes. Significant reduction of parasitic load after immunization confirms that, in sheep, the intranasal vaccination with DGNP with total extract of antigens (TE), (DGNP/TE), provided significant protection against latent toxoplasmosis and subsequent transplacental transmission.

## Goat vaccines

A few studies have been performed that involve the immunization of goats against *T. gondii* infection. In two studies, goats were immunized with *Hammondia hammondi* and *Hammondia heydorni* parasites, which were examined for their efficacy as vaccine candidates against congenital toxoplasmosis. In a study does were immunized with 10^6^ oocysts of nonpathogenic coccidiym *H. hammondi* 17–23 days before breeding, and between 51 and 119 days, does were orally challenged with 1,000 infective oocysts of the GT-1 strain of *T gondii* (51) (Table 1). It was found that 5 out of 6 vaccinated goats produced healthy kids within the expected date of gestation, whereas unvaccinated dams aborted at 17 days of infection. During post-mortem examinations, it was found that the unvaccinated does also had a history of retaining the placenta. These results confirmed *H. hammondi* as a promising vaccine candidate to protect against *T. gondii* infection and prevent abortion in goats (51). In a subsequent study by Dubey (52), goats were immunized with *H. hammondi* or *H. heydorni* (Table 1) and challenged with 1,000 or 100,000 oocysts of strain GT-1 of *T. gondii*. The survival rate was found to be higher among those vaccinated with *H. hammondi* (4 out of 4) than among those vaccinated with *H. heydorni* (1 out of 4). Antibody production was low (*H. hammondi-*vaccinated group) or undetectable (*H. heydorni*-vaccinated group). In another study, 1 month prior to breeding, goats were experimentally immunized with a live vaccine, S48 strain (Ovilis Toxovax^®^), which was approved for use in sheep, then challenged with 10,000 oocysts at 90 days of gestation. The immunized group showed a lower abortion rate than the unimmunized group, indicating the efficacy of strain S48 against congenital toxoplasmosis (50) (Table 1).

## Summary

Toxoplasmosis has both public health concerns and economic significance, especially in the case of goat and sheep farming worldwide. In 2012, according to a report published by the advisory committee on the microbiological safety of food standards agency, human *Toxoplasma* infection was the second leading cause of death from foodborne illness in the United States, accounting for 24% of all deaths compared with 28% for *Salmonella* spp. (73), and the consumption of meat from sheep was responsible for 30% of human illnesses (7). Effective vaccination appears to be the solution to control this parasite. A range of vaccination strategies has been developed for use in sheep and goats, including live, killed, DNA, and nano-particle-based vaccines. Vaccination with live-attenuated vaccines is reported to induce a significant immune response in sheep and goats, although the risk of a live vaccine reverting to its virulent parental form should not be overlooked. The only authorized vaccine for ovine use is ToxoVax™ (Animal Health Ltd., New Zealand), which has been implemented in a few countries (53) and provides partial protection for ewes (74). However, this vaccine can induce some adverse effects, has limitations such as a short shelf-life, and cannot be used in humans.

Over the last few decades, trials in ovine with subunit vaccines have failed to protect against *Toxoplasma* infection. A previous study using surface antigens of *T. gondii* in conjunction with ISCOMs for immunization failed to show efficacy (36). In another study, sheep were immunized intranasally with *T. gondii* tachyzoites extract (TE) in PLG micro- and nano-particles along with cholera toxin as an adjuvant, but despite the development of humoral and cellular immune responses, protection was not evident (37). DNA vaccines elicit distinct humoral and cellular immune responses (42, 46, 47) but are accompanied by a number of concerning safety issues, such as genomic incorporation of the plasmid causing activation of oncogenic proteins and antibody production against the vaccine DNA itself (74). Therefore, different adjuvants and nano-carriers have been employed as cargo to deliver the DNA vaccines (75). Recombinant subunit vaccines or virus-like particles are safe for use, but do not elicit the same immune-stimulating effects as DNA vaccines and live attenuated vaccines, are extremely costly to manufacture, and involve extensive purification steps (76).

For the vaccination of sheep and goats, recombinant vaccines offer an attractive option for the development of a new vaccine. IFN-γ-producing CD4^+^ and CD8^+^ T cells are found in efferent lymph after ovine are infected with *T. gondii*, and a protective Th1 immune response develops over time (77). Moreover, the production of IFN-γ and IL-12 in the early stage of infection (8 days post-infection) has also been reported by Verhelst et al. (78), which aids the host's defense mechanisms. These immune processes play a crucial role in protecting against infection, especially during pregnancy. Ovine toxoplasmosis is, therefore, a significant problem in veterinary and public health because of the partial effectiveness of current vaccines. The researchers are trying to mine out an effective vaccine against toxoplasmosis. The goal of immunization should prevent this zoonotic, the worldwide spread of toxoplasmosis.

## Conclusion

The intricacy of the life cycle of *T. gondii* with numerous developmental stages, and the various routes of infection, make the development of an effective vaccine challenging. Most studies to date have been performed in sheep, with few being carried out in goats; however, goats are one of the most important animals susceptible to toxoplasmosis, and undercooked goat meat (containing tissue cysts) and unpasteurized goat milk (containing tachyzoites) present a risk of transmission to humans. More attention should therefore be paid to goats in future research. To develop an effective vaccine, future studies should focus on discovering more potent, antigenic parasitic antigens that can trigger protective immunity by inducing cellular and humoral immune responses against challenge infection and will be able to prevent abortion and vertical transmission. In addition, vaccines could be developed that contain multiple antigens such as DNA vaccine pools, and immunogenicity could be boosted by employing novel delivery systems (e.g., vectors, adjuvants, nano-particles). Antigens that are involved in migration, invasion, replication, and other events in the development of parasites also require further investigation as potential immunizing antigens. The aim of vaccination should be to control congenital, acute, and chronic infections in small ruminants, with the outcome being safer meat for human consumption and reduced environmental contamination with oocysts.

## Author contributions

Conceptualization, validation, supervision, and funding: YN. Reviewing and editing: YN and TH. Original draft preparation: TH. All authors contributed to the article and approved the submitted version.

## Funding

This study was supported in part by the Research Program on Emerging and Re-emerging Infectious Diseases [21fk0108137h (YN)] from the Agency for Medical Research and Development (AMED), KAKENHI Grants from the Japan Society for the Promotion of Science [20KK0152 (YN)].

## Conflict of interest

The authors declare that the research was conducted in the absence of any commercial or financial relationships that could be construed as a potential conflict of interest.

## Publisher's note

All claims expressed in this article are solely those of the authors and do not necessarily represent those of their affiliated organizations, or those of the publisher, the editors and the reviewers. Any product that may be evaluated in this article, or claim that may be made by its manufacturer, is not guaranteed or endorsed by the publisher.
